# Neural Processes Underlying Tool Use in Humans, Macaques, and Corvids

**DOI:** 10.3389/fpsyg.2020.560669

**Published:** 2020-09-23

**Authors:** María J. Cabrera-Álvarez, Nicola S. Clayton

**Affiliations:** Department of Psychology, University of Cambridge, Cambridge, United Kingdom

**Keywords:** tool use, neural mechanisms, neural network, causal reasoning, macaques, corvids

## Abstract

It was thought that tool use in animals is an adaptive specialization. Recent studies, however, have shown that some non-tool-users, such as rooks and jays, can use and manufacture tools in laboratory settings. Despite the abundant evidence of tool use in corvids, little is known about the neural mechanisms underlying tool use in this family of birds. This review summarizes the current knowledge on the neural processes underlying tool use in humans, macaques and corvids. We suggest a possible neural network for tool use in macaques and hope this might inspire research to discover a similar brain network in corvids. We hope to establish a framework to elucidate the neural mechanisms that supported the convergent evolution of tool use in birds and mammals.

## Introduction

The classic definition of tool use in animals is “the use of an external object as a functional extension of mouth or beak, hand or claw, in the attainment of an immediate goal” (van Lawick-Goodall, 1970, p.195), following the observations of tool use in a wild population of chimpanzees (Goodall, 1964). Since then, the definition of tool use has evolved to encompass the physical properties of tools, specifying that a tool must alter “the form, position or condition of another object, another organism or the user itself” (Beck, 1980, p.10), and to include ways of manipulating and manufacture of tools (St Amant and Horton, 2008) in order to distinguish between those species that have the capability to create or use an external object to solve a problem via dynamic mechanical interactions, i.e., “flexible tool users,” and those that are “stereotyped tool users,” i.e., species that perform object-related mechanical actions that are not intended to have a goal-directed interaction with another object (Hunt et al., 2013). This is an important distinction because flexible tool use is not phylogenetically widespread and seems to require a certain level of cognitive processing (Baber, 2003). In this paper, our focus will primarily be on flexible tool use.

The definition of tool use specifies that the tool has to be detached from the substrate and directly held in the animal’s hand or mouth. This definition has opened a debate in the field of tool-use since many animals, including birds, cannot hold tools in their hand or mouth but instead use their beak or foot and others may throw or drop objects to achieve their goals (St Amant and Horton, 2008). Given these differences in the way tools are used, a distinction was made between “true tool-users,” i.e., those that follow the traditional definition of tool-use albeit broadening the criteria to include beaks and limbs [e.g., New Caledonian crows (*Corvus moneduloides*) using twigs as hooks to retrieve food from small holes (Hunt, 1996)], and borderline or “proto-tool” users, i.e., those that use objects to obtain food that would otherwise be out of reach but do not hold these objects in their limbs or mouths/beaks [e.g., American crows (*Corvus brachyrhynchos*) dropping nuts in roads to get them crashed by the passing of automobiles over them (Grobecker and Pietsch, 1978)]. From a cognitive perspective, it makes sense to make this distinction, since holding the tool in a part of the user’s body might make the user include that object as part of their own body, while those animals that just throw or drop objects might not have the ability to include the object in their body-image. As Jacob Bronowski graciously expressed, “the hand is the cutting edge of the mind” (Bronowski, 1975, p.116), and thus we should not forget their importance for body awareness.

Recently, Fragaszy and Mangalam (2018) developed a theory of tool use which the authors termed “tooling.” This theory is framed in biomechanical and spatial concepts of action in order to determine when an object is used as a tool. It aims to reconceptualize the phenomenon of tool use. The authors developed the concept of “tooling,” which we adopt as a legitimate description of what we consider tool use: “Tooling is deliberately producing a mechanical effect upon a target object/surface by first grasping an object, thus transforming the body into the body-plus-object system, and then using the body-plus-object system to manage (at least one) spatial relation(s) between a grasped object and a target object/surface, creating a mechanical interface between the two” (Fragaszy and Mangalam, 2018, p.194).

Before Goodall’s observations, it was widely believed that tool use was a uniquely human characteristic (Oakley, 1972), since the use and manufacture of tools has historically been linked to the emergence of technical intelligence in humans given the complex problem solving and planning needed to create and use composite tools (i.e., tools made of two or more joined parts) (Ambrose, 2001). Since then, many observations of both proto- and true-tool use have been reported, not only in primates but also in other mammals (Mann et al., 2008; Root-Bernstein et al., 2019), birds (Hunt, 1996), reptiles (Dinets et al., 2015), fish (Brown, 2012), and insects (Pierce, 1986). It is worth mentioning that flexible tool use is mostly found in birds and primates, while insects and fish mostly show stereotypical tool use (Hunt et al., 2013). Most significantly, Hunt made the remarkable discovery that New Caledonian crows manufacture and craft a variety of tools which they use to obtain food that cannot be reached in any other way (Hunt, 1996). Subsequent research by Hunt and other members of Gray and Taylor’s research groups have revealed many fascinating findings about the complexity of physical cognition in these birds (Hunt and Gray, 2004; Taylor et al., 2009, 2010). These observations provide evidence that evolutionarily distant species are capable of similar complex motor skills that require a certain level of cognitive ability to perform them.

Hodos (1987) suggested the study of animal tool use as one of the specific intellectual abilities that can be used as a proxy to understanding the concept of animal intelligence proposed by Macphail (1987). Hodos (1987) argued that we would understand animal intelligence more rapidly if we focus our efforts in the study of specific intellectual abilities rather than in the search of general intelligence. However, our understanding of the neural processes underlying tool use in non-human animals remains scant, even though descriptive reports and ecological literature related to animal tool use has grown dramatically.

Having a proper understanding of the neural mechanisms underlying tool use is pivotal to comprehend the evolutionary processes that enabled evolutionarily distant animals to achieve similar cognitive capabilities because the comparison of the brain structures that are needed for this specific intellectual ability will shed light on the evolutionary paths that give rise to animal intelligence. This review compiles information regarding brain areas active during tool use in humans and macaques, and will suggest possible areas in the bird brain that could be a focus of study in the future.

## Tool Use in Humans

The neural basis of tool use in humans was first studied in patients with brain lesions that impaired their ability to use tools, a consequence of a disorder known as apraxia (Johnson-Frey, 2004; Lewis, 2006; Higuchi et al., 2007; Frey, 2008). Patients with apraxia do not show difficulties in linguistic, sensory or lower level motor functions. However, they do exhibit an impaired ability to carry out acquired skills, including, although not specific to, the use of tools. There are two types of apraxia that affect tool use: ideomotor apraxia and ideational, or conceptual, apraxia. In ideomotor apraxia, although patients know what to do with a tool and can grasp and manipulate it, they seem to be unable to represent the associated motor actions needed to properly use the tools, failing to pantomime how the tools are used. These patients suffer from damage to the left posterior parietal and/or premotor cortex, or damage to the corpus callosum that results in isolation of the left hemisphere from the right (Frey, 2008). On the other hand, in ideational or conceptual apraxia, patients know how to handle the tools, but can not follow the order of a sequence of movements to achieve a goal that is the product of a multistep action. Ideational apraxia patients commonly have lesions at the intersection of the temporal-parietal-occipital cortices of the left hemisphere (Frey, 2008). The studies of apraxia show, not only that the motor skills and conceptual knowledge about complex actions such as tool use are dissociable, but also that they are represented in dissociable neural systems within the left cerebral hemisphere (Johnson-Frey, 2004; Lewis, 2006; Frey, 2008).

Subsequently, fMRI and PET studies in healthy humans highlighted the areas of the cortex that are active during tool use or during mimicking and imaging tool use (reviewed in Lewis, 2006, see his Figure 5B). Lewis’ figures (2006) show that most activity during tool use is in the left hemisphere of the human cortex. This is the case for right-handed people, while the right hemisphere might have higher activation during tool use in left-handed people (reviewed in Lewis, 2006). Further studies comparing right- vs. left-handed people are needed in order to disentangle the lateralization of tool use in humans.

**FIGURE 1:**
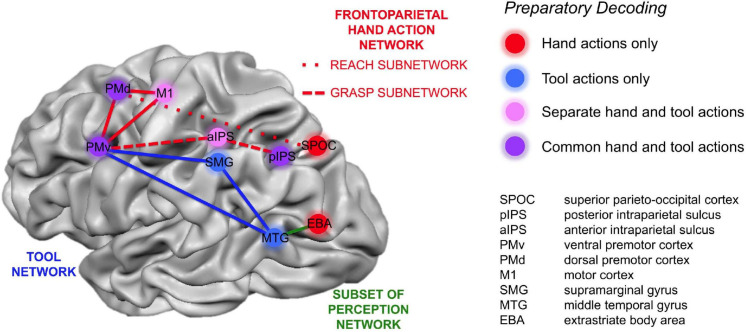
Cortical areas that coded only hand actions (red), only tool actions (blue), both hand and tool actions but using different neural representations (pink), and areas coding an action independently of it being performed with the hand or a tool (purple). Reproduced from Gallivan et al. (2013).

**FIGURE 2:**
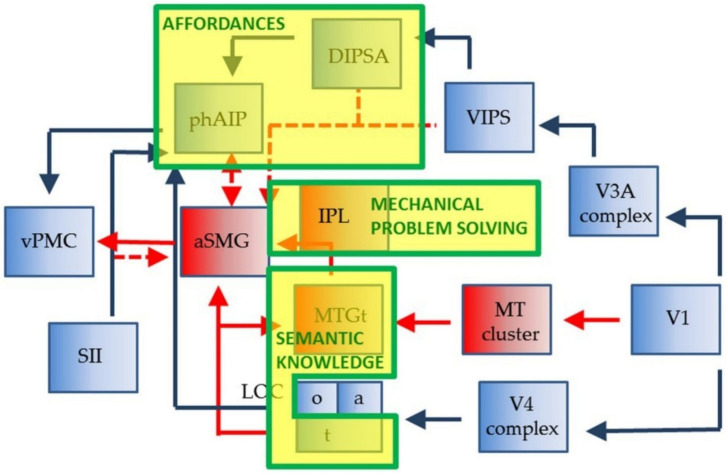
Representation of the human brain network suggested to be involved in tool use, including biological actions in blue, tool actions in red and blue, and cognitive processes in yellow boxes with green outlines. Dashed lines represent postulated connections. Reproduced from Orban and Caruana (2014).

Peeters et al. (2009, 2013) identified the anterior supramarginal gyrus (aSMG), a specific region of the human brain left inferior parietal lobule (IPL), as being involved in both the execution and observation of tool actions. They did not find a similar activation in the IPL of rhesus monkeys that were trained to use tools. In a subsequent study, Gallivan et al. (2013) found specific brain areas involved only in tool-related actions, in contrast with brain regions involved only in hand-related actions, and suggested a brain network for human tool use (Figure 1). This brain network was later expanded to include the connection between the aSMG, which is active during the observation of the tool being moved to achieve a goal, and the putative human homolog of anterior intraparietal (phAIP) in macaques, a region active during observation of tool grasping (Figure 2; Orban and Caruana, 2014). Figure 2 showcases the cognitive processes involved in tool use, which are reasoning affordances (i.e., forming conclusions about the qualities of an object that defines its possible uses), mechanical problem solving (i.e., finding solutions to novel mechanical problems), and semantic knowledge (i.e., a type of long term memory consisting of concepts, facts, ideas, and beliefs). This suggested brain network also highlights the brain areas underpinning these cognitive processes, which would all provide input to the aSMG. For further reading, additional reviews on human tool use have been recently published (Osiurak and Badets, 2016; Reynaud et al., 2016).

Although the cerebellum is not mentioned in these brain networks, its function in tool-use has been under debate despite its role in sensory-motor control and learning of complex action sequences. For example, it is unclear why cerebellar lesions do not cause ideomotor apraxia if the cerebellum stores representations of tool-use skills (Johnson-Frey, 2004). Yet, its development during human evolution and its interactions with the neocortex have been related to greater computational efficiency for dealing with increasingly complex cultural and conceptual environments (Weaver, 2005). In great apes, cerebellar volume and lateral asymmetry of the cerebellum is related to species-specific differences in performance and hand preference for task that require precise motor skills, such as tool use and aimed throwing (Cantalupo and Hopkins, 2010). Additionally, the cerebellum plays an important role in the evolution of the capacity for planning, execution and understanding of complex behavioral sequences—including tool use and language (Barton, 2012; Barton and Venditti, 2014), and the neural mechanisms of tool use may be precursors for the neural basis of language and abstract thought (Hihara et al., 2003; Iriki and Taoka, 2012; Steele et al., 2012). These authors have suggested that the use of tools as equivalents of body parts would have triggered the development of more advanced problem solving skills: abstract thinking (i.e., the ability to think about things that are not physically present) was essential for the development of conceptual thinking (i.e., the ability of integrating a series of features that group together to form a class of ideas or objects) and hence, the development of language.

Despite the relevance of tool use to human evolution and the benefits that its study would bring to our understanding of brain evolution, the neural processes underlying tool use in other animals have not yet been closely studied, partly due to the methodological difficulty in reproducing the same sort of studies in animals. Given the hypothesis that tool use in humans would have led to the evolution of more complex cognitive abilities, comparisons of both the cognitive and neural mechanisms of tool use in humans and other vertebrates would increase our understanding of the evolution of physical cognition. An obvious starting point for such a comparison is to focus on non-human primates as there are many examples of tool-use in the wild, such as axing oysters (Gumert et al., 2009), nut-cracking, ant-dipping, and termite fishing, which have been previously reviewed (e.g., van Schaik et al., 1999; Whiten et al., 1999; Emery and Clayton, 2009), and in captivity, even in species that have not been observed using tools in their natural environment (van Lawick-Goodall, 1970; van Schaik et al., 1999). It is interesting to note that despite assumptions that non-human primates show flexible tool use, high cognitive abilities may not be necessary for the performance of tool use in many of the examples given (Penn and Povinelli, 2007). Instead, simpler forms of learning, such as affordance learning (i.e., learning about the use or purpose that an object can have, either directly or through social learning) may be responsible in some species for instrumental object manipulation (Whiten et al., 1999; Martin-Ordas et al., 2008; Gruber et al., 2011).

Keeping in consideration that the development of human high order cognition, including abstract thinking, might have been enhanced once humans used tools as equivalents of body parts (Iriki and Taoka, 2012), it is reasonable to assume that the comparison between humans and other vertebrates’ cognitive and neural mechanisms of tool use would increase our understanding of the evolution of physical cognition. Despite the abundant number of tool-use observations and cognitive studies, the study of the neural mechanisms governing tool-use in non-human primates is challenging. PET scanning studies, for example, require primates to remain completely immobile, except for the limb that uses the tool, during data collection to prevent confounding motion artifacts. Accurate measurements can only be achieved by confining the limbs not involved in the studied actions to small spaces and by limiting movement of the subject’s head using a custom-made chair. These constraints explain why there is so little non-human research on the matter, and also why most studies have very small sample sizes.

## Tool Use in Macaques

Macaques are of particular importance because they have special neurons that become active both when they see another individual performing an action and when they do the action themselves (Rizzolatti and Craighero, 2004). These neurons, located in premotor cortex F5, are of two types: canonical neurons and mirror neurons. Canonical neurons respond to the presentation of a graspable object or are active when the macaques grasp that object, while mirror neurons respond when the macaque sees object-directed actions (Rizzolatti and Craighero, 2004; Iriki, 2006; although see Hickok, 2010 for a critical review on mirror neuron function). Similar neurons were subsequently found in both humans and birds (Rizzolatti and Craighero, 2004; Welberg, 2008), although it remains unclear whether they exists in birds outside of the context of song learning. The ability to use tools and the presence of mirror neurons in their brains make macaques an interesting model for the study of tool use in vertebrates since mirror neurons are active during object-directed actions. Although there are neurocognitive studies exploring tool-use in a number of other species of non-human primates (e.g., Hopkins et al., 2012, 2017; Phillips and Thompson, 2013; Mayer et al., 2019) we have focused the following section on macaques because our objective is to suggest a possible neural network for tool use in a non-human primate species. For this purpose, using a single genus instead of a combination of findings from multiple species prevents us from generating a misleading network, since different species might differ in many ways, including anatomically, mechanistically, behaviorally, and cognitively.

### Macaque Active Brain Areas During Tool Use

Obayashi et al. (2001, 2002, 2004, 2007) performed a series of studies on two awaken-behaving male Japanese monkeys (*Macaca fuscata*). They explored the brain areas that are active during tool use by using PET scans during a task in which the subjects were previously trained to use tools to collect an unreachable food pellet. They used a control task in which the subjects experienced almost the same sensorimotor circumstances as in the experimental task, but without any learning involved (i.e., manipulation of the control apparatus did not result in the macaques learning how to reach the reward, but once the macaques had manipulated the control apparatus the experimenters moved the reward within their reach). In their 2001 study, the subjects had to reach the pellet with one rack. In their 2002 study, they had to poke a pellet with a rack out of a transparent tube and reach it with a second rack. In their 2004 and 2007 studies, the subjects had to obtain an unreachable pellet by manipulating a joystick or a pair of dials, respectively, which controlled the position of a shovel that moved in a two-dimensional space. They found the following active brain areas during tool use in macaques (Obayashi et al., 2001, 2002, 2004, 2007).

#### Prefrontal Cortex (PFC)

Specifically, area 9/46 seems to be involved in executive functions, since it is active during a sequence of tool combination tasks but not during single tasks (Obayashi et al., 2002). It is also active during abstract actions like remote operations using dials in a set of sequences (Obayashi et al., 2007). Together with the cerebellum, this area is involved in the automatization of learned motor sequences (Obayashi et al., 2004).

#### Intraparietal Sulcus (IPS)

This region is the area of the brain that creates, stores, and updates the body-image, i.e., the primate’s awareness of where its limbs are in space, and what actions they are performing (Obayashi et al., 2001, 2002, 2004, 2007). It has an important role in tool use because it provides the individual with an updated spatial representation of the situation, which is vital for the successful completion of the goal.

#### Inferior Temporal Cortex

Including the posterior portion of inferior temporal cortices (area TEO). This region is involved in object recognition and memory. Its extensive connections with IPS suggests that it might help this other area in maintaining and manipulating the body-image (Obayashi et al., 2002).

#### Premotor Cortex

There are two areas of interest within this region: F5 and dorsal premotor cortex (PMD). F5, the area containing mirror neurons in macaques, is involved in the execution of goal-directed manual actions (Rizzolatti and Craighero, 2004). The PMD is involved in planning coordinated activation of muscles and joints to accurately perform desired movements (Obayashi et al., 2001, 2004). Thus, the combined activation of these two areas might be involved in the accurate execution of goal-directed actions performed with tools.

#### Pre-supplementary Motor Area (pre-SMA)

This area, which receives input from the premotor cortex (especially from F5), was suggested to be involved in the maintenance and updating of the body-image, which would be helpful for the execution of tool-based/use actions and sequential movements (Obayashi et al., 2001, 2004).

#### Basal Ganglia

It was suggested that the basal ganglia, as well as the IPS bimodal neurons, is involved in the creation and maintenance of the spatiotemporal representation of the hand during tool use (Obayashi et al., 2001).

#### Cerebellum

The cerebellum was suggested to be involved in the learning processes required for tool use and “reconstruction of the acquired body-image,” and “may modulate higher cognitive functions of the executive process as a cerebro-cerebellar loop from an anatomical perspective” (Obayashi et al., 2007).

These brain areas are important for tool use but that is not their only role. They can also be involved in less functional or less goal-directed forms of object manipulation, such as object exploration or object play, often claimed to be associated in the development, evolution and daily expression of tool use (Smith, 1982; Kerney et al., 2017).

**FIGURE 3:**
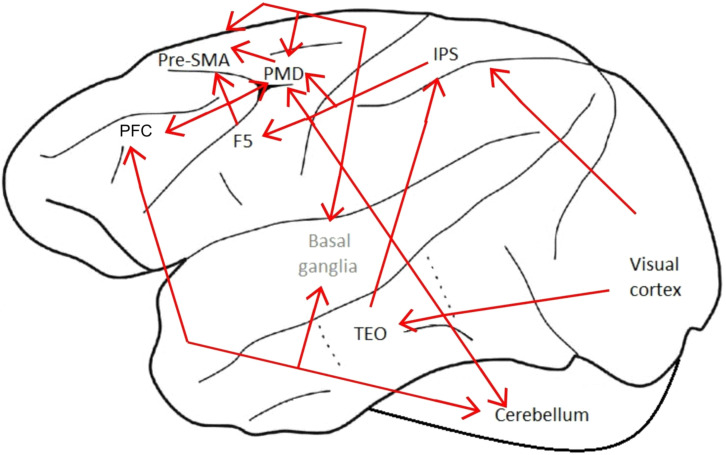
Suggested macaque brain network for tool use. Suggested pathways in red. The names of cortical areas are in black, and subcortical areas in gray. The location of the areas is approximated. IPS, Intraparietal Sulcus; F5, Area F5 of the Premotor Cortex; PFC, Prefrontal Cortex; PMD, Dorsal Premotor Cortex; Pre-SMA, Pre-Supplementary Motor Area; TEO, Posterior Portion of the Inferior Temporal Cortex.

Based on the information collected in the aforementioned studies and the available data on macaque brain connections (Schaal, 1999; Hihara et al., 2006; Van Essen and Dierker, 2007; Borra et al., 2008; Smaers et al., 2013; Takada et al., 2013; Rizzolatti et al., 2014), we suggest the following brain network for tool use in macaques, represented in Figure 3. The visual input about the tool and the task or problem that needs to be solved using that tool is processed in the visual cortex, which sends this information to the inferior temporal cortex and the IPS. The inferior temporal cortex would process the information related to object recognition, and would then send this information to the IPS. All of this information would be processed in the IPS and a spatial representation of the situation would be created. This information would be sent to area F5 and PMD. These two areas would coordinate the muscles to accurately perform the goal-directed action, and would send this information to the Pre-SMA, which is involved in the execution of sequential movements. The Pre-SMA would update the basal ganglia about the motor action and the basal ganglia would update the Pre-SMA and the PMD about the hand movements during tool use. The information would be sent to a PFC-basal ganglia-cerebellum network, involved in novel motor sequences learning and automatization of learned motor sequences. Finally, the cerebellum would ensure a coordinated motor action. It will be interesting to know whether the same brain regions are involved in these aspects of tool use in other animals or whether they are specific to the macaque brain. Additionally, it will be interesting to study the correlation between the specific patterns of brain activity and the motor movements involved, including in the correlation the levels of cognitive and behavioral control, such as distinctions between flexible and stereotypic tool use, and true versus proto tool use. An exciting first step would be to evaluate these issues in corvids, since like macaques and some other primates, these birds have relatively large brains for their body size, are highly social, have relatively long life spans and are known to use tools for extractive foraging and other problem solving tasks (Emery and Clayton, 2004a) including species that only do so in captivity (Clayton and Emery, 2015). Indeed Clayton and her colleagues have argued for the convergence of cognition in primates and corvids (e.g., Emery and Clayton, 2004b; Seed et al., 2009; Taylor and Clayton, 2012; Legg et al., 2017; Baciadonna et al., 2020).

## Tool Use in Corvids

The study of the neural processes of tool use in birds is of great interest, given that some species of birds have shown cognitive abilities and tool-use capability similar to those seen in primates (van Lawick-Goodall, 1970; Lefebvre et al., 2002; Emery and Clayton, 2004a, b, 2009; Clayton and Emery, 2015;, although see a comparison between chimpanzees and New Caledonian crows’ tool use in McGrew, 2013). By studying the neural processes of tool use in birds we could identify the brain areas involved in meticulous motor skills and complex cognitive abilities such as problem solving and future planning, which would help us understanding the complexity of the avian brain and its analogies with the mammalian brain (Grodzinski and Clayton, 2010). This is crucial to increasing our understanding of the convergent evolution of physical cognition among vertebrates.

Among birds, corvids are the family with the highest known number of true tool-user species (Lefebvre et al., 2002). For instance, New Caledonian crows manufacture hook tools out of plants to collect food in the wild (Hunt, 1996), with different populations within New Caledonia showing different strategies to manufacture these tools, which is hypothesized to have evolved through a process of cumulative change (Hunt and Gray, 2003). New Caledonian crows also select tools of the right length to achieve their goal (Chappell and Kacelnik, 2002), shape unfamiliar materials to create usable tools for specific tasks (Weir et al., 2002), manufacture tools of the best diameter to achieve a reward (Chappell and Kacelnik, 2004), can infer weights from how objects move under a current of wind (Jelbert et al., 2019), and are capable of spontaneous meta-tool use (using a tool to obtain a tool in order to achieve a goal) (Taylor et al., 2007). Recently, it was found that Hawaiian crows (*Corvus hawaiiensis*), which were considered extinct in the early 2000s and currently live in captivity, are capable of tool use and tool manufacture (Rutz et al., 2016).

Other species of corvids that are not known to be tool-users in the wild are capable of readily using and making tools in captivity. For example, rooks (*Corvus frugilegus*) were able to spontaneously create hook tools from pieces of straight wire and use them to collect food that would otherwise have been out of beak reach (Bird and Emery, 2009a). Furthermore they could use stones to raise the water level to reach a floating reward (Bird and Emery, 2009b), a task also performed by other non-tool-using corvids such as Eurasian jays (*Garrulus glandarius*) (Cheke et al., 2011). Northern blue jays (*Cyanocitta cristata*) also made and used tools to obtain food out of reach (Jones and Kamil, 1973). Furthermore, ravens (*Corvus corax*) demonstrated problem-solving skills by string-pulling to collect food (Heinrich and Bugnyar, 2005), and are capable of planning for events using tools with delays of up to 17 h, including bartering tokens for food rewards (Kabadayi and Osvath, 2017). Many of these behaviors are under cognitive control, as tasks such as wire bending, meta-tool use, inferring the weight of objects, using objects to raise volumes of water, and bartering for tools, require some level of mental processing in order to conceive the problem, to understand the characteristics of the available objects, to plan a solution, and to successfully perform the task to solve the problem. Unfortunately, the neural mechanisms underlying these mental processes are still unknown.

It is for all these reasons that corvids make an excellent model for the study of the neural mechanisms of tool use (Clayton and Emery, 2015; Güntürkün, 2020). Furthermore, they belong to the order Passeriformes, in which the neural mechanisms of song learning in songbirds has been extensively studied, and so there is a wider amount of available information on neuroanatomy and neural networks for corvids compared to other families of tool-user birds (Jarvis et al., 2005). For example, some species of the order Psittaciformes (which includes parrots, lorikeets, and cockatoos) are capable of using and manufacturing tools (Auersperg et al., 2012, 2014; Lambert et al., 2015), and, like corvids, they are also social, big-brained, and vocal learners. However, psittaciformes are evolutionarily less closely related to songbirds, and so the study of their neural circuits has received less attention.

Lefebvre et al. (2002) found that the brain size of true tool-users was significantly larger than that of borderline tool-user species. However, the relatively larger brain of crows and ravens is not correlated to tool use, innovative feeding strategies, and dispersal success (Jønsson et al., 2012). Lefebvre et al. (2002) also found that, after examining the size of four different areas of the telencephalon, the neostriatum [later renamed as nidopallium (Jarvis et al., 2005)] was the only area significantly larger in true tool-using birds compared to borderline tool-users. However, this size difference does not mean that the nidopallium is the only area involved in tool use; rather, it means that the cognitive ability to use tools might depend on the relative size of the nidopallium.

Subsequent studies showed that the neuronal density of the brain of several bird species significantly exceeds the neural densities of many mammals, including primates, of similar brain mass (Olkowicz et al., 2016). This finding could explain why birds, which have relatively small brains compared to mammals, are capable of performing high cognitive tasks. When comparing studies of apes, corvids and pigeons, Güntürkün et al. (2017, p.39) suggest that a “neuronal surplus may translate into faster and more flexible learning, making the acquisition of certain abstract abilities a much easier task”. However, a fundamental difference between avian and mammalian brains is that birds lack pyramidal neurons. The inability to create long extensions (i.e., association fibers) that arise from pyramidal neurons means that to exhibit similar behavior to mammals, birds would need more neurons to make the same computations. Additionally, the small size of avian neurons also allows them to have higher neuronal density. An additional hypothesis that we suggest is that the nuclei-organized bird forebrain, unlike the cerebral cortex of primates which is organized in layers, might be computationally more powerful to encode tool-use skills than the isolated neurons in monkeys. To explain the differences in tool use between humans and monkeys, Orban and Caruana (2014) suggested that humans are capable of using tools because we have grouped neurons that respond to tool action observation, unlike some species of monkeys which were unable to learn to use tools. This same hypothesis can therefore be applied to birds, as we have done here. However, further studies are needed to figure out whether tool-use birds own specific neurons that respond to tool action observation.

In mammals, the prefrontal cortex plays a crucial role in problem solving (Mushiake et al., 2009). Given the many examples of problem-solving skills of corvids (e.g., Hunt, 1996; Chappell and Kacelnik, 2002, 2004; Weir et al., 2002; Clayton, 2007; Taylor et al., 2007; Bird and Emery, 2009a, b; Tebbich et al., 2012), it is reasonable to assume that there must be a bird brain area capable of processing information to solve problems in a similar fashion as the mammalian prefrontal cortex does (Güntürkün, 2005; Güntürkün and Bugnyar, 2016). Identifying this possible area is crucial to understand how the cognitive abilities of corvids and apes have evolved via convergent evolution. A specific part of the nidopallium, called the caudolateral nidopallium (NCL) has been suggested as an analogous to the mammalian prefrontal cortex due to its high density of dopaminergic axons (Divac and Mogensen, 1985; Waldmann and Güntürkün, 1993; Güntürkün, 2005) and their function in reward processing (Koenen et al., 2013), prospective processing (reviewed in Colombo et al., 2017), reversal learning, response inhibition and working memory, obtained from studies in pigeons (reviewed in Striedter, 2013). The involvement of NCL in tool use is uncertain, as neurological studies in tool-using birds are rare. However, given the abundant connections of the NCL with other brain areas involved in tool use, it is thought that the NCL is “a critical integrative area for telencephalic sensorimotor pathways” (Striedter, 2013, p.63). Figure 1 in Striedter (2013) highlights the major avian brain areas and circuit diagrams that emphasize the role of NCL in tool use. Neurocognitive studies in crows have shown the involvement of the NCL in cognitive tasks that are important for tool use, such as visual working memory (Veit et al., 2014), associative learning (Veit et al., 2015), abstraction of general principles (Veit and Nieder, 2013), or relative numerosity discrimination (Ditz and Nieder, 2015, 2016). The NCL also shows properties such as flexible neuronal tuning depending on behaviorally relevant tasks (Veit et al., 2017) which is crucial to encode task relevant information.

New Caledonian crows, which are known for their exceptional ability to build and use tools, have an enlarged mesopallium, pallidostriatal complex, septum and tegmentum, compared to three other passeriformes (carrion crows, jays and sparrows) (Mehlhorn et al., 2010). Mehlhorn et al. (2010) suggest that the mesopallium might be required for enhancement of basic tool skills, while the nidopallium, which was also enlarged in this species although not significantly, might have a role in cognitive and motor skills required for basic tool use. They also suggest that the pallidostriatal complex might be important in these birds to learn to manufacture and use tools in novel and familiar situations, and the tegmentum would be involved in the fine motor skills needed for tool manufacture and use, while the septum would integrate several stimuli in order to modulate complex behaviors, which might not be directly involved with tool use. In summary, the study by Mehlhorn et al. (2010) corroborates the findings of previous studies, such as Timmermans et al. (2000), in that the size of the mesopallium is correlated to the feeding innovation rate in birds. However, it is difficult to determine what roles the septum and tegmentum could play in tool use without further investigations, particularly for the tegmentum, which is a large, multifunctional region.

Another region that seems to be important for tool use is the cerebellum. The cerebellum seems to be active during tool use in macaques and humans, as described previously. In birds, it was found that, although the total size of the cerebellum was not significantly different between tool-user and non-tool-user species, the number of folds in the cerebellar cortex was significantly larger in the former (Iwaniuk et al., 2009). It is possible that the increase in the number of folds might have been a way to supply the increased motor, sensory, and cognitive processing demands of the cerebellum of tool-user birds. Furthermore, a recent study in parrots describes a telencephalic-midbrain-cerebellar circuit that resembles the one in primates (Gutiérrez-Ibáñez et al., 2018), which is associated with the evolution of complex cognitive abilities, as described in previous sections. Particularly, the medial spiriform nucleus (SpM), which connects the pallial regions of the telencephalon with the cerebellum in birds, is greatly enlarged in parrots compared with other birds, suggesting that a stronger link between the pallium and cerebellum is associated with cognition (Gutiérrez-Ibáñez et al., 2018). Further studies in birds are needed to clarify the specific role of the cerebellum and the SpM during tool use.

The current literature differs in whether tool-using birds learn to use tools from conspecifics or not. A study on woodpecker finches (*Cactospiza pallida*) found that these birds probably learn to use tools by trial and error during their development (Tebbich et al., 2001). However, a study of Goffin cockatoos (*Cacatua goffiniana*) found social transmission of tool use and tool manufacture in the males of this species (Auersperg et al., 2014), and a study of New Caledonian crows found evidence for probable transmission of tool design between crows (Hunt and Gray, 2003). Thus, although true imitation of tool use has not been observed in birds and, hence, it is not possible to claim that tool-user birds must have mirror neurons, it is important to keep in mind that some of the tool-user species have developed the cognitive abilities to learn specific motor actions by observing others. This feature may imply the development of specific neural characteristics in tool-user birds, which might not be present in species that do not use tools and do not learn from others. In order to answer whether birds have developed neural mechanisms that are similar to humans and non-human primates when learning to use tools, it would be of great interest to study the activity in neurons of the above mentioned brain areas during observations of tool use. However, the size of these brain areas is so large that more research needs to be done before specific nuclei within these areas can be selected for study. The study of the neural processes of tool use in birds is therefore of pivotal interest in order to answer this and other questions, such as what mechanisms are behind the neural development that allows the generation of the same kind of complex behavioral patterns in unrelated species, so we can establish a framework to elucidate the neural mechanisms that supported the convergent evolution of tool use in birds and mammals.

## Conclusion

The study of the neural processes underlying tool use in humans and non-human primates has received increased attention over the last decade. Early studies in patients suffering from apraxia showed that complex actions that require motor skills and conceptual knowledge, such as tool use, are represented in dissociable neural systems within the left cerebral hemisphere. Two interconnected regions are particularly important, the anterior supramarginal gyrus and the putative human homolog of the anterior intraparietal cortex. However, it is clear that these two regions do not work alone, and a complex neural network is required to use tools. Similarly, in macaques there are also two areas of particular interest: area F5 and the dorsal premotor cortex, both located within the premotor cortex. However, although these two regions seem to be of utmost importance during tool use in macaques, it is evident that, as in humans, tool use requires the activation of a complex network of brain activation, as we have suggested here. We hope that a similar brain network can be elucidated for tool-using corvids in the future, given the abundant evidence of tool use and manufacture in this family of birds. We know that New Caledonian crows and Hawaiian crows can use and manufacture tools, and New Caledonian crows can infer their physical properties (Hunt, 1996; Chappell and Kacelnik, 2002, 2004; Weir et al., 2002; Taylor et al., 2007; Rutz et al., 2016; Jelbert et al., 2019). Furthermore, corvid species that are not tool-users in the wild, such as rooks, Eurasian jays, and northern blue jays, can use and manufacture tools in captivity and use these skills to solve problems (Jones and Kamil, 1973; Seed et al., 2006; Tebbich et al., 2007; Bird and Emery, 2009a, b; Cheke et al., 2011). Additionally, ravens can use tools to obtain unreachable food and use tokens to barter (Heinrich and Bugnyar, 2005; Kabadayi and Osvath, 2017). Despite this plethora of examples, we have yet to discover the neural mechanisms underpinning these behaviors and their cognitive control in corvids. However, we do know about the neural mechanisms of song production and song learning in songbirds, which are birds of the same order as corvids. The knowledge on songbird neuroanatomy may serve as a basis to explore the neural mechanisms of tool use in corvids and to elucidate a neural network underpinning tool-using behavior. We have reviewed the current evidence of several bird brain regions that could be involved in tool use in corvids and that should be the focus of study in future research, such as the NCL, mesopallium, pallidostriatal complex, SpM, cerebellum, and areas of the tegmentum. Understanding the neural processes of tool use in animals other than primates would not only increase our understanding of the evolution of physical cognition in vertebrates, including a better understanding of animal intelligence, but also benefit our society by providing new models with which scientists can work to understand the origins of complex motor skills, and ultimately improve the lives of those affected by motor disabilities.

## Author Contributions

MC-Á wrote the manuscript, with critical reviews and important discussions and additions by NC. Both authors contributed to the article and approved the submitted version.

## Conflict of Interest

The authors declare that the research was conducted in the absence of any commercial or financial relationships that could be construed as a potential conflict of interest.
